# Decreased 25-Hydroxyvitamin D Levels in Patients With Vestibular Neuritis

**DOI:** 10.3389/fneur.2019.00863

**Published:** 2019-08-08

**Authors:** Yunqin Wu, Zhizhou Hu, Minyan Cai, Zhenyi Fan, Weiwei Han, Qiongfeng Guan, Min Zhou, Li Li, Wang Yan, Xiaoxiong Lu

**Affiliations:** ^1^Department of Neurology, Hwa Mei Hospital, University of Chinese Academy of Science, Ningbo, China; ^2^Department of Neurology, LongYan First Hospital, Affiliated to Fujian Medical University, Longyan, China; ^3^Department of Neurology, Zhuji People's Hospital of Zhejiang Province, Shaoxing, China; ^4^Department of Rehabilitation, Hwa Mei Hospital, University of Chinese Academy of Science, Ningbo, China

**Keywords:** vestibular neuritis, vertigo, 25-hydroxyvitamin D, vitamin D deficiency, inflammation

## Abstract

**Objective:** Vestibular neuritis (VN) is characterized by acute onset of vertigo, nausea, and vomiting, without auditory or other neurological symptoms. Although the pathogenesis of VN is not yet clear, many studies have shown that a pro-inflammatory environment can lead to the induction and progression of the disease. Considering the importance of vitamin D in modulating the activation, proliferation, and differentiation of inflammatory physiological processes, we hypothesized that decreased serum vitamin D may be associated with the development of VN. In this study, we evaluated serum levels of 25-hydroxyvitamin D [25(OH)D] in patients presenting acutely with VN and healthy controls and investigated the possible correlation of serum 25(OH)D levels with VN.

**Methods:** A total of 59 consecutive patients diagnosed with VN within 7 days of symptom onset and 112 age- and sex-matched healthy controls referred to Hwa Mei Hospital, University of Chinese Academy of Science, between March 2017 and March 2019 were recruited. Demographic and clinical data, such as age, sex, height, weight, living habits, ongoing health problems, and medication history, for all subjects were recorded, and levels of 25(OH)D were measured and compared.

**Results:** Serum levels of 25(OH)D were lower in patients with VN than in controls (19.01 ± 6.53 vs. 22.94 ± 6.74 ng/ml, *p* < 0.001). Patients with VN had a higher frequency of vitamin D deficiency (61.0 vs. 34.8%, *P* = 0.001) than did controls. Regression analyses demonstrated that vitamin D deficiency was associated with VN, with an odds ratio of 4.53 (95% CI = 1.342–15.279, *P* = 0.015).

**Conclusion:** This prospective study is the first to evaluate serum 25(OH)D levels in patients with VN and found that decreased serum 25(OH)D may be associated with VN occurrence.

## Introduction

Vestibular neuritis (VN) is a common cause of peripheral vestibular disorder and is characterized by acute onset of vertigo, nausea, and vomiting, without auditory or other neurological symptoms (1). VN is a benign condition that is generally restricted to one attack, with only a small number of patients having repeated attacks during their lifetimes, and almost complete recovery is expected within 6 months of onset (2, 3). The incidence of VN is ~3.5 to 15.5 cases per 100,000 people (4).

At present, various hypotheses, such as viral infection, vascular ischemia, and immunological mechanisms, have been suggested as causes of VN, but the exact etiology and pathogenesis of this condition remain unknown (5). Accumulating evidence supports the idea that inflammation plays an important role in the induction and progression of VN. It has been reported that the levels of acute inflammatory response markers such as fibrinogen, D-dimers, and C-reactive protein as well as mean neutrophil-to-lymphocyte and platelet-to-lymphocyte ratios are significantly elevated in VN patients during episodes compared to healthy subjects (6–8). Moreover, patients with VN show significant elevations in the percentage of pro-inflammatory CD40^+^, TNF-α^+^, COX-2^+^, or CD38^+^ peripheral blood mononuclear cells in comparison to healthy controls (9).

Vitamin D (VitD) is a fat-soluble vitamin that regulates calcium and phosphate levels in the circulation and plays an important role in modulating inflammatory and immune processes (10, 11). Epidemiological studies have indicated that VitD deficiency is associated with the occurrence of vestibular system disease, such as benign paroxysmal positional vertigo (BPPV) (12–15) and Meniere's disease (MD) (16). In addition, a few reports have described a beneficial effect of vitamin D supplementation in preventing attacks of BPPV (17, 18) and MD (16).

Building on the background of these studies, we hypothesized that low serum vitamin D may be associated with the development of VN. In this prospective study, we measured and compared serum levels of 25-hydroxyvitamin D3 [25(OH)D] in VN patients and healthy controls and collected pilot data to test our hypothesis.

## Materials and Methods

### Patients and Study Design

All patients who were diagnosed with VN at the Department of Neurology, Hwa Mei Hospital, University of Chinese Academy of Science, from March 2017 to March 2019 and 112 age- and sex-matched healthy controls were enrolled in this prospective study. The diagnostic criteria for VN (1, 5) were as follows: (1) acute vertigo attack, accompanying nausea, vomiting, or postural instability; (2) horizontal spontaneous nystagmus with a rotational component toward the unaffected ear and vestibular dysfunction confirmed by the video head impulse test; (3) absence of hearing loss, tinnitus, and any other neurological signs; and (4) normal magnetic resonance images of the cerebellum and brain stem. We also recorded data, such as age, sex, height, weight, ongoing health problems, medication history, and past and present of history smoking and drinking, for all subjects. Due to the pilot nature of this investigation, we excluded as many as possible influencing factors, and subjects with a history of vertigo or imbalance were excluded. The following exclusion criteria were applied: (1) non-cooperation; (2) long-term steroid therapy, vitamin D supplementation or osteoporosis treatment; and (3) any systemic or chronic disease, such as chronic renal failure, chronic liver disease, or hormonal disorders influencing VitD results. As nausea and vomiting during the acute period may affect serum vitamin D levels in patients with VN, we excluded 25(OH)D values for these VN patients when the interval between the onset of symptoms and blood collection was more than 1 week.

The study was approved by the ethics committee of Hwa Mei Hospital, University of Chinese Academy of Science (protocol number KY-2017-014-02). The study adhered to the principles of the Declaration of Helsinki. Written informed consent was obtained from all subjects.

Fasting early-morning venous blood was collected from all subjects. Complete blood cell counts, total serum cholesterol, high-density lipoprotein, low-density lipoprotein, and cholesterol triglyceride were quantified by standard methods at the laboratory of Hwa Mei Hospital, University of Chinese Academy of Science. Serum 25(OH)D was measured using an API3200 liquid chromatography-mass spectrometer/mass spectrometer system (Applied Biosystems, Foster City, CA). According to the internal standard, the 25(OH)D level was classified as normal (30 ng/ml), insufficient (20 to <30 ng/ml), or deficient (<20 ng/ml) (19).

### Clinical Evaluation of Vestibular Neuritis

All patients were hospitalized at the Department of Neurology and received symptomatic treatment for dizziness such as dimenhydrinate, metoclopramide, and diazepam. The video head impulse test (vHIT) was used for all VN patients for recording in each direction of the semi-circular canal (GN Otometrics, Denmark). To ensure accuracy and consistency, we defined vHIT gain values <0.8 in the horizontal canal and <0.7 in the vertical canal as an abnormal reflex (20, 21).

### Statistical Analysis

Continuous variables are expressed as the mean ± SD, and categorical variables are described as numbers and percentages. The Kolmogorov-Smirnov test was used to assess the data distribution. An unpaired *t*-test and a chi-square test were employed to determine differences between groups. Multiple logistic regression analysis was performed to estimate the odds ratio (OR) for the association between VN and relevant covariates. Statistical analyses were performed using SPSS 22.0 (SPSS Inc., Chicago, IL, USA). All *P* < 0.05 were considered indicative of statistical significance.

## Results

### Demographics and Clinical Characteristics of the Subjects

A total of 74 patients were diagnosed with VN at our institution from March 2017 to March 2019. Among them, six patients who rejected the 25(OH)D test and four patients who took hormone therapy or medications for osteoporosis or had chronic liver dysfunction or renal failure were excluded. In addition, five patients were eliminated from the study because the interval between the onset of symptoms and hospitalization was more than 1 week. In total, 59 VN patients, including 36 women (age range = 22–79 years, mean ± SD = 53.9 ± 15.5 years) and 23 men (age range = 29–87 years, mean ± SD = 55.1 ± 15.1 years), met the inclusion criteria. The interval between the onset of symptoms and initial evaluation varied from 1 h to 6.8 days (median = 2.0); blood collection varied from 10 h to 7 days (median = 2.6), and 69.5% of blood was collected within 3 days of the symptom onset. There were 41 cases with horizontal semi-circular canal function loss, 13 cases with posterior semi-circular canal function loss, and five cases with both. No significant differences in age distribution, the sex ratio, body mass index (BMI), comorbidities, or lifestyle were found between the two groups (*P* > 0.05; Table 1).

**Table 1:** Demographic and biochemical characteristics of vestibular neuritis patients and controls.

	**VN (*n* = 59)**	**Control (*n* = 112)**	***P***
Age (years)	54.39 ± 15.25	54.07 ± 14.86	0.895
Sex (M/F)	23/36	45/67	0.627
BMI (kg/m^2^)	24.07 ± 3.42	23.51 ± 3.11	0.278
Hypertension [*n* (%)]	19 (32.2%)	30 (26.78%)	0.592
Diabetes [*n* (%)]	7 (11.87%)	12 (10.71%)	0.749
Smoking (%)	17 (28.81%)	29 (25.89%)	0.846
Drinking (%)	10 (16.95%)	21 (18.75%)	0.834
Total cholesterol (mmol/l)	4.69 ± 0.89	4.76 ± 1.09	0.694
LDL-C (mmol/l)	2.57 ± 0.86	2.64 ± 0.73	0.661
HDL-C (mmol/l)	1.39 ± 0.52	1.38 ± 0.36	0.886
Triglycerides (mmol/l)	1.33 ± 0.84	1.61 ± 0.97	0.074
25-Hydroxyvitamin D (ng/ml)	19.01 ± 6.53	22.94 ± 6.74	<0.001
Normal	4 (6.8%)	18 (16.1%)	
Insufficiency	19 (32.2%)	55 (49.1%)	
Deficiency	36 (61.0%)	39 (34.8%)	

*BMI, body mass index, as defined as weight in kilograms divided by the square of height in meters; LDL-C, low-density lipoprotein cholesterol; HDL-C, high-density lipoprotein cholesterol. Values are expressed as the n (%) or mean ± SD. P values were calculated using an independent t-test or the chi-squared test. The differences were considered significant at P < 0.05*.

### Serum 25(OH) D Levels and Risk of VN

Mean serum 25(OH)D levels in VN patients were significantly lower than in healthy controls, both for men (20.07 ± 6.07 vs. 23.85 ± 7.08 ng/ml, *p* = 0.033) and women (18.33 ± 6.79 vs. 22.31 ± 6.48 ng/ml, *p* = 0.004). The frequency of vitamin D deficiency was 61.02% (36/59) among VN patients, which was significantly higher than that among healthy controls (34.82%) (39/112) (*P* = 0.001). Multiple logistic regression analyses adjusted for age, sex, BMI, the presence of diabetes and hypertension, lipid profiles, smoking, and drinking demonstrated that 25(OH)D deficiency was associated with VN, with an OR of 4.53 (95% CI = 1.342–15.279, *P* = 0.015; Table 2).

**Table 2:** Multiple logistic regression analysis to identify independent risk factors for vestibular neuritis.

**Variables**	**OR (95% CI)**	***P***
Age	0.98–1.025	0.831
BMI	0.971–1.213	0.147
25-Hydroxyvitamin D		
Insufficiency 20 to <30 ng/ml	0.455–5.453	0.473
Deficiency <20 ng/ml	1.342–15.279	0.015

*BMI, body mass index: the body mass index is the weight in kilograms divided by the square of the height in meters. Multiple logistic regression analysis included age, sex, BMI, the existence of smoking, and drinking, diabetes, hypertension, lipid profiles*.

## Discussion

Several studies have reported the association of vestibular disorders with vitamin D deficiency; however, there are no pilot data on serum vitamin D levels in VN patients. In this study, we investigated levels of serum 25(OH)D in patients with VN and healthy controls and found that levels were lower in patients with VN compared with in healthy controls. Moreover, the proportion of 25(OH)D deficiency was higher in patients with VN than in healthy controls, and 25(OH)D deficiency may be a risk factor for VN.

There is increasing evidence for an inflammatory component in the progression and etiology of VN that might be the result of various infections. A large-scale epidemiological study has found that preceding or concurrent infectious illnesses occur in 43 to 46% of VN cases (4). Acute inflammatory response biomarkers such as serum D-dimer, C-reactive protein, and fibrinogen levels are reportedly significantly higher in VN patients than in healthy subjects (6, 7). Additionally, novel markers of systemic inflammation, such as the neutrophil-to-lymphocyte ratio and platelet-to-lymphocyte ratio, are significantly higher in VN patients than in controls (8), and the ratio of thymus lymphocyte subsets (CD4 T-helper/CD8 T-suppressor cells) in peripheral blood is elevated in 48% of patients with VN (22). Furthermore, Kassner et al. demonstrated that patients with acute VN exhibit significantly elevated serum levels of C-reactive protein and percentages of pro-inflammatory CD40^+^, tumor necrosis factor-α (TNF-α), cyclooxygenase-2 (COX-2), or CD38^+^ positive inflammatory activation in peripheral blood mononuclear cells compared to healthy individuals (9). Moreover, VN patients treated promptly with corticosteroids showed a better clinical outcome compared to placebo, whereas virostatics alone did not improve recovery (23, 24). All of these findings suggest that elevation of the inflammatory component may contribute to VN induction and progression.

VitD is a secosteroid hormone that is mainly synthesized in the skin and then undergoes hydroxylation in the liver and kidney to generate biologically active 1,25-dihydroxyvitamin D3 [1,25(OH)_2_D_3_] (10). Binding of 1,25(OH)_2_D_3_ to nuclear vitamin D receptor (VDR) produces a complex that acts as a transcription factor that regulates multiple downstream pathways involved in proliferation, differentiation, and immunomodulation (11, 25). Among these processes, the anti-inflammatory effect of vitamin D has attracted extensive attention. Indeed, 1,25(OH)_2_D_3_ exerts an anti-inflammatory effect on the inflammatory profile of cells, downregulating expression and production of pro-inflammatory cytokines, including TNF-α, IL-1β, IL-6, and IL-8, and upregulating anti-inflammatory cytokines, such as IL-4, IL-5, and IL-10 (26–30). All these functions are mainly mediated through nuclear VDR (nVDR). Previous studies have found that nVDR is distributed in multiple cells of the human immune system, such as Treg cells, neutrophils, dendritic cells, B lymphocytes, and macrophages, as well as in the inner ear, epithelium of the crista ampullaris, membranous semi-circular canal, and surrounding osteocytes (31–33). Considering the presence of VDR in the inner ear, it seems logical that vitamin D might affect vestibular and hearing function. For example, vitamin D receptor-deficient (VDR^−/−^) mice show balance dysfunction when evaluated using accelerating rotarod, tilting platform, rotating tube, and swim tests (34).

Several studies have reported the important role of vitamin D in normal vestibular function in humans. Sanyelbhaa et al. reported abnormal ocular and cervical vestibular-evoked myogenic potentials in subjects with vitamin D deficiency, suggesting that vitamin D deficiency results in the production of abnormal otoconia (35). In addition, epidemiological studies have indicated that low vitamin D levels are associated with the development of BPPV and MD and that supplementation with vitamin D may reduce further attacks in those with BPPV and MD (12–18).

In this study, we found that low levels of 25(OH)D may be a risk factor for VN occurrence. The key question regards how vitamin D is involved in VN pathogenicity. We think the answer may be related to the function of vitamin D in regulating inflammatory and immune responses. The leading hypothesis involves reactivation of a latent neurotropic virus in VN. The characteristic histopathological features are atrophy of the vestibular nerve and vestibular sensory epithelium, similar to the histopathological findings for known viral diseases, such as herpes zoster (36, 37). Recently, *in vivo* work has demonstrated that herpes simplex virus (HSV) infection can induce VN and sudden deafness in a mouse model (38). Herpes simplex virus type 1 DNA has also been detected in human vestibular ganglia at the time of autopsy. These viruses (herpes simplex virus types 1 and 2 and herpes zoster virus) have a propensity for invading sensory neurons, establishing latency within the nucleus of ganglion cells and becoming reactivated under some condition (36, 39, 40). The local inflammatory reaction of the inner ear caused by prior or concurrent infection may hypothetically be regulated/inhibited by normal levels of vitamin D, similar to suppression of the production of pro-inflammatory cytokines in patients with congestive heart failure (41) and in experimental bowel disease (42). When the level of vitamin D decreases, the inhibitory adaptive immune system and inflammatory function may be weakened. Under such conditions, the amount of pro-inflammatory mediators may increase, leading to reduced microvascular perfusion of the vestibular organ and causing nerve swelling, entrapment, and loss of function. Considering the anti-inflammatory effect of vitamin D and the presence of VDR in the inner ear, it seems logical that disturbance of the level of this vitamin is involved in this disease. Nonetheless, further study is needed to clarify the specific role of vitamin D in the pathogenesis of VN.

This study is the first to explore serum 25(OH)D levels in patients with VN. We found that reduced serum 25(OH)D was associated with the occurrence of VN. Our study had some limitations. First, the present study was conducted at a single center with a relatively small sample size; in particular, only healthy controls were included, and vitamin D levels with a non-vestibular patient control group, which might limit the generalizability of the results, were not compared. Second, in this prospective study, we did not detect inflammatory factors or perform follow-up to determine vitamin D levels at different stages of disease, which prevented us from drawing firm conclusions. Recent reports indicate that mediators of inflammation are significantly elevated in patients with VN compared to healthy subjects. Further studies at multiple centers with large sample sizes and dynamic assessments of vitamin D levels and mediators of inflammation are needed to clarify these issues, possibly using animal models to provide more robust evidence for the role of vitamin D in the potential pathogenesis of VN.

## Conclusion

In summary, the present study revealed significantly lower serum vitamin D levels in patients with VN compared to those in healthy controls, and vitamin D deficiency was associated with VN occurrence. As the involvement of vitamin D deficiency in the underlying pathophysiology of VN remains uncertain, further studies are needed to investigate the molecular mechanisms that occur in this condition and to perhaps suggest a novel therapeutic target.

## Data Availability

The raw data supporting the conclusions of this manuscript will be made available by the authors, without undue reservation, to any qualified researcher.

## Ethics Statement

The study was approved by the ethics committee of Hwa Mei Hospital, University of Chinese Academy of Science (protocol number KY-2017-014-02). The study adhered to the principles of the Declaration of Helsinki. Written informed consents were obtained from all subjects.

## Author Contributions

YW, XL, and ZH conceived and led the work. YW, MC, ZF, MZ, WH, QG, LL, WY, and XL reviewed and acquired the patients' medical record. YW, WY, and MZ performed the statistical analysis of the data. YW and XL wrote the article.

### Conflict of Interest Statement

The authors declare that the research was conducted in the absence of any commercial or financial relationships that could be construed as a potential conflict of interest.
